# Severe symptomatic hyperparathyroidism—Is it carcinoma?—Case report and literature review

**DOI:** 10.1002/ccr3.2886

**Published:** 2020-06-22

**Authors:** Catarina A. Pereira, Susana Garrido, Cláudia Amaral, Olinda Lima, Helena Cardoso

**Affiliations:** ^1^ Endocrinology Department Centro Hospitalar e Universitário do Porto Porto Portugal; ^2^ Anatomopathology Department Centro Hospitalar e Universitário do Porto Porto Portugal

**Keywords:** hypercalcemia, parathyroid carcinoma, primary hyperparathyroidism

## Abstract

Parathyroid carcinoma is a rare disease, difficult to diagnose and associated with a poor prognosis. It must be suspected preoperatively, based on clinical and imaging grounds, in order to perform the best surgical option and avoid compromising patient's prognosis.

## Graphical abstract

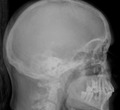

## 1 INTRODUCTION

Parathyroid carcinoma is a rare entity, usually associated with a poor outcome, requiring more aggressive surgery than parathyroid adenomas. We here review preoperative factors that suggest parathyroid carcinoma in the presence of primary hyperparathyroidism, based on a case report of a patient with severe hypercalcemia and preoperative suspicion of parathyroid carcinoma, not confirmed by histopathology.

Parathyroid carcinoma is a rare disease, usually associated with a poor prognosis.^1^, ^2^, ^3^ In face of severe primary hyperparathyroidism, especially if associated with kidney and bone disease, one must think about the possibility of parathyroid carcinoma.^4^, ^5^ Other clinical and imaging features, such as high plasma levels of calcium and parathyroid hormone (PTH) and tumor size, also favor the possibility of parathyroid carcinoma preoperatively.^4^, ^6^ It is essential to identify this group of patients before surgery in order to proceed to a more aggressive surgical resection. The most suitable surgery for these patients is *en bloc* resection,^4^ with removal of the ipsilateral thyroid lobe,^7^ as this strategy proved to improve prognosis.^8^, ^9^

## 2 CASE REPORT

We present the case of a 34‐year‐old man, diagnosed with primary hyperparathyroidism after a pathologic right subtrochanteric fracture.

The patient had high blood pressure, diagnosed at the age of 32, well controlled with an angiotensin II receptor antagonist, and a past history of moderate alcohol consumption (completely abstinent since the age of 30). He had also an episode of acute lithiasic pancreatitis at age 30, at the time attributed to alcohol consumption. The patient had been complaining for at least 4 years of bone pain, muscle weakness precluding autonomous walking, polydipsia, polyuria, and constipation. A depressive humor, associated with mental and behavioral disturbances, was also evident, with serious implications on his daily life routines. Despite this, no medical investigation had ever been made.

**Figure 1:**
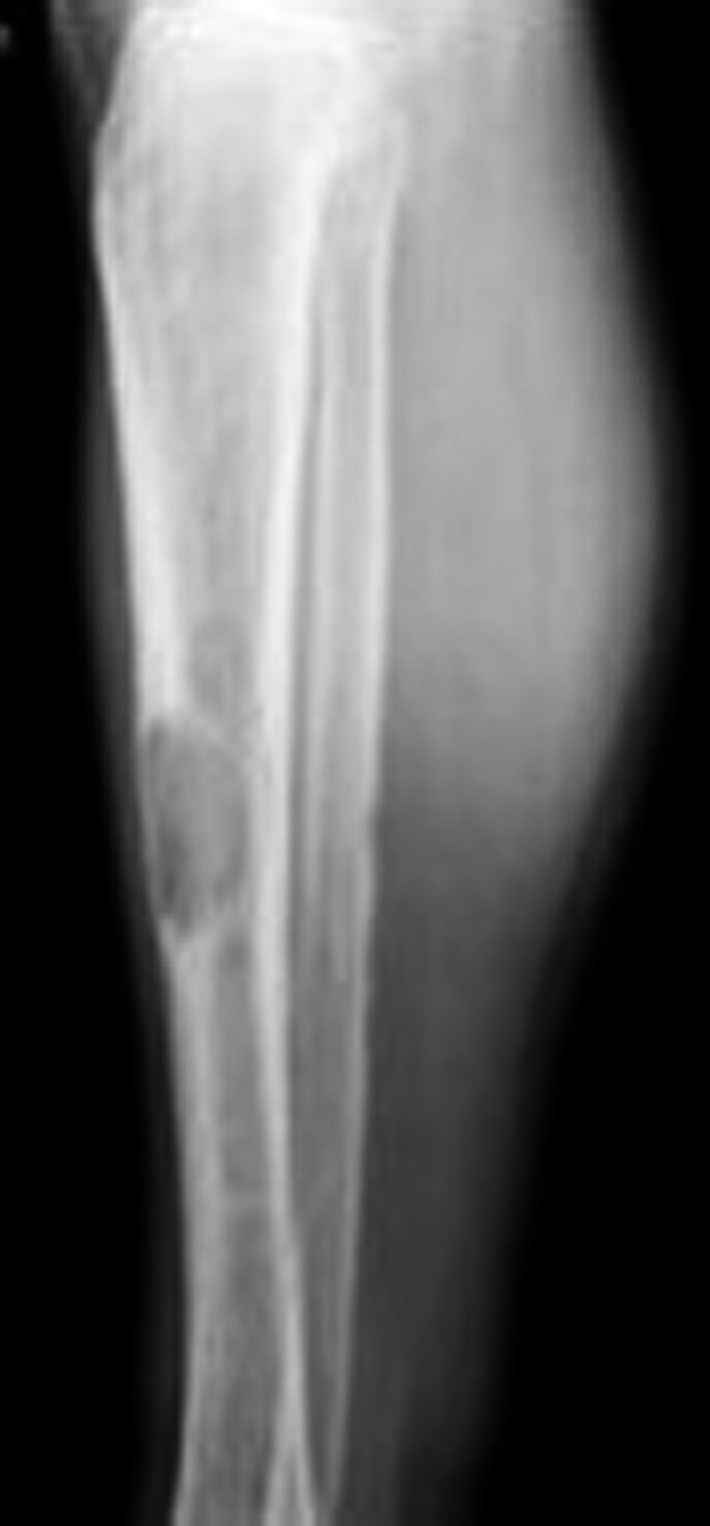
Right lower limb radiography—well‐defined lithic lesion, with sparse reactive bone, corresponding to brown tumor of tibia

**Figure 2:**
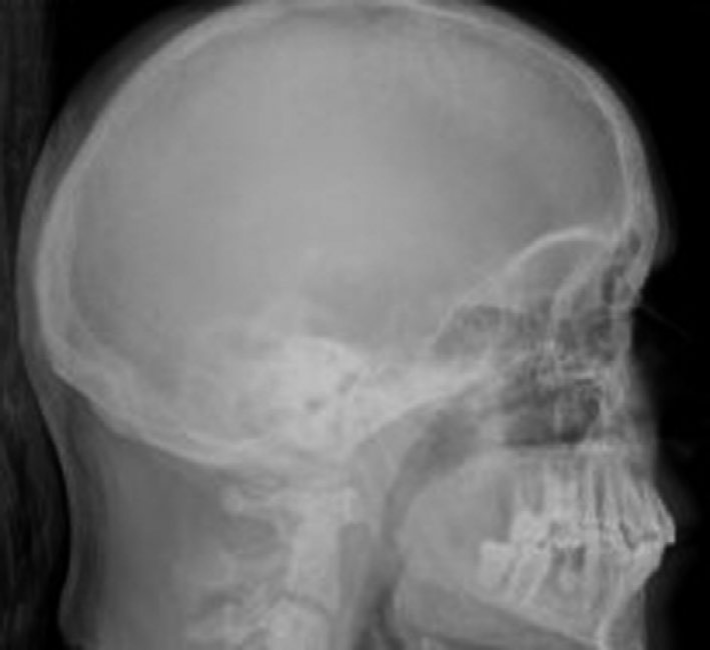
Skull radiography—“salt and pepper” sign

**Figure 3:**
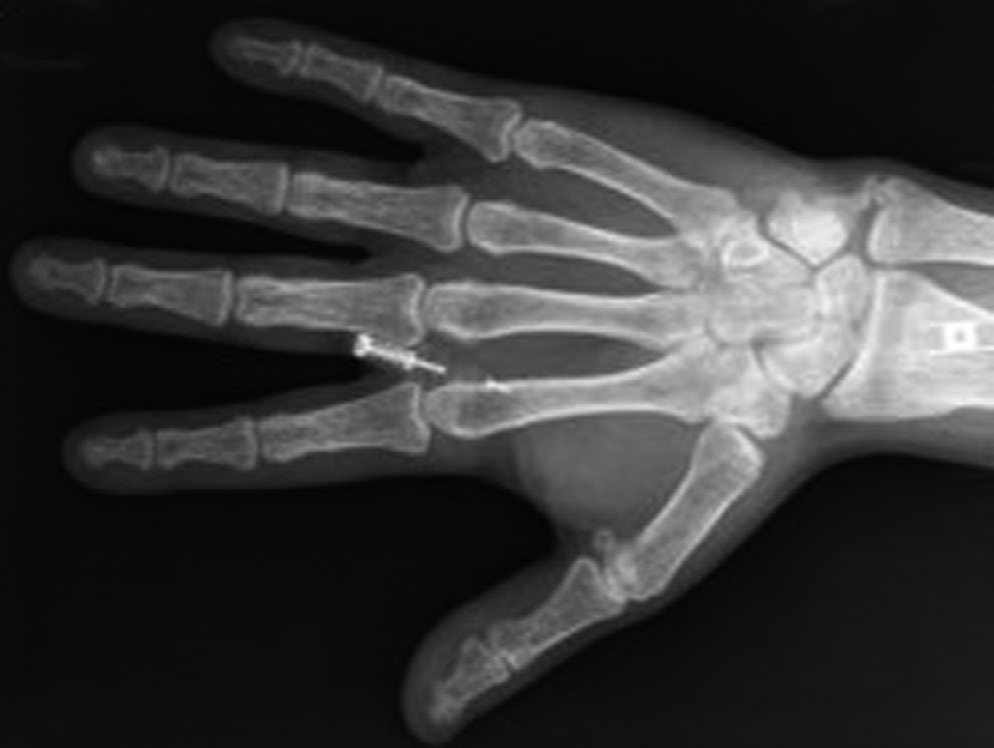
Hand radiography—diffuse bone rarefaction with subperiosteal reabsorption in radial side of second and third fingers' phalanges

The patient presented in the Emergency Department in 2012 with a pathologic right subtrochanteric fracture. The study of phosphocalcic metabolism, requested in this context, showed a severe PTH‐dependent hypercalcemia, with a serum albumin‐adjusted total calcium of 4.39 mmol/L (normal range, 2.09‐2.42 mmol/L) and PTH of 3000 pg/mL (normal range 15‐65 pg/mL). Phosphorus levels were of 0.65 mmol/L (normal range, 0.87‐1.45 mmol/L), magnesium 0.62 (normal range, 0.60‐1.10 mmol/L), 25(OH)D 29.15 nmol/L (normal range, >70 nmol/L), and total alkaline phosphatase 1065 U/L (normal range, 32‐104 U/L). He had no family history of hypercalcemia or endocrine tumors. Exuberant bone disease was documented, namely osteitis fibrosa cystica and a brown tumor, involving the right tibia (Figure 1). Skull radiography had the typical “salt and pepper” sign (Figure 2), and hand radiography showed a diffuse bone rarefaction with subperiosteal reabsorption in the radial side of the second and third fingers (Figure 3). The patient also had chronic kidney disease stage 3b (plasmatic creatinine 2.0 mg/dL, glomerular filtration rate 42 mL/min/1.73 m^2^), as well as renal lithiasis, nephrocalcinosis, and presumed nephrogenic diabetes insipidus. No neck masses were palpable.

Neck ultrasound showed a nodular image with 26 mm in parathyroid topography, highly vascularized and lateralized to the left, and ^99m^Tc‐sestamibi scintigraphy documented an increased uptake in the same location. Bone scintigraphy showed no evidence of metastasis.

After initial treatment with vigorous endovenous fluid therapy and a single dose of 60 mg endovenous infusion of pamidronate, the patient underwent left inferior parathyroidectomy, performed without complications. Intraoperative PTH decreased from 2621 to 282 pg/mL 10 minutes postexcision and normalized to 57 pg/mL at the fourth postoperative day. Macroscopically, the removed parathyroid measured 2.5 cm in the largest dimension and had a pink coloration and elastic consistency, with no signs of adherence to the contiguous structures. Microscopic examination revealed an epithelial neoplasia with trabecular pattern, low mitotic index, without necrosis and surrounded by a thin fibrous capsule on the periphery. Immunohistochemistry staining was positive for calcitonin and chromogranin and negative for synaptophysin and thyroglobulin. Immunohistochemistry for parafibromin was not performed. Surgical resection was complete, although marginal. The lesion was classified as a parathyroid neoplasm of uncertain malignant potential or atypical adenoma (based in the criteria described in Table 1)^10^.

**Table 1:** Histological criteria of carcinoma in parathyroid neoplasias^34^

Absolute criteria of malignancy (the presence of any one of the following criteria is sufficient for a diagnosis of malignancy)
1. Invasion into surrounding structures: thyroid esophagus nerves, or soft tissues
2. Histologically documented regional or distant metastasis

Features associated with malignancy (in the absence of the absolute criteria, at least 2, preferably 3 or more, of the following features have to be present in order to establish a diagnosis of malignancy)
1. Capsular invasion
2. Vascular invasion
3. Readily identifiable mitotic figures (>5 per 10 high‐power fields)
4. Broad intratumoral fibrous bands splitting the parenchyma and separating expansile nodules
5. Coagulative tumor necrosis (has to be distinguished from infarction, which can occur in parathyroid adenoma)
6. Diffuse sheet‐like monotonous small cells with high nuclear/cytoplasmic ratio
7. Diffuse cellular atypia
8. Macronucleoli present in many tumor cells
For the rare cases that show some but inconclusive features of malignancy, the designation “atypical parathyroid adenoma” may be applied

Postoperatively, the patient developed “hungry bone” syndrome, needing endovenous calcium during the first postoperative days and oral calcium supplementation for 15 months. All symptoms resolved after achieving normocalcemia, and a subjective cognitive improvement was also evident, which enabled the patient to autonomously perform his daily activities and walk without assistance. Diabetes insipidus also resolved and renal function improved (plasma creatinine 1.2 mg/dL, glomerular filtration rate 78 mL/min/1.73 m^2^). Genetic testing for mutations in the CDC73 (HRPT2) gene was negative; MEN‐1 mutations were not evaluated due to the absence of evidence of other associated tumors or familial history of hypercalcemia or MEN‐1‐associated tumors.

At his last follow‐up visit at the Endocrinology Outpatient Clinic (3 years postoperatively), the patient was asymptomatic and maintained normal serum calcium and PTH levels (albumin‐adjusted total calcium 2.26 mmol/L and PTH 57.2 pg/mL). There was no evidence of uptake in ^99m^Tc‐sestamibi scintigraphy 1 year postsurgery, and bone scintigraphy did not show any lesion suggestive of metastasis. Bone mineral density was normal in femoral neck and spine, but still had criteria of osteoporosis in the forearm (T score −3.4) 4 years postoperatively.

## 3 DISCUSSION

We here report a case of high clinical preoperative suspicion of parathyroid carcinoma and discuss its relevance in the operative management of this entity.

Parathyroid carcinoma is a rare endocrine neoplasia, with an incidence of approximately 11 cases per 10 million persons per year,^11^, ^12^ corresponding to less than 1% of all cases of primary hyperparathyroidism.^6^, ^13^

The pathogenesis of parathyroid carcinoma is still not well known, but is probably related with the interaction of several genetic and environmental factors.^6^ Most cases are sporadic, but some familial cases have been described,^6^, ^13^ mainly in the context of hyperparathyroidism‐jaw tumor syndrome (HPT‐JT), an autosomal dominant inherited endocrine neoplasia syndrome associated with a high prevalence of malignancy.^14^, ^15^ The mutated gene is the HRPT2/CDC73, which codifies the protein parafibromin.^16^ This gene is also frequently mutated in sporadic cases of parathyroid carcinoma.^17^ Other somatic mutations potentially implicated in parathyroid carcinoma are those affecting RB and p53 genes, although a clear pathological meaning was still not found. Despite the description of some cases of parathyroid carcinoma after neck irradiation or long‐lasting secondary hyperparathyroidism,^18^ there is no evidence of an associated malignant transformation.^19^, ^20^

Parathyroid carcinomas often occur at a younger age than seen in adenomas; unlike adenomas, that are more common in female patients, carcinomas have an equal distribution between genders.^21^

In most cases, diagnosis is made during surgery or at histological analysis. Sometimes though, it is only made years after surgical resection, when hypercalcemia relapses due to local recurrence or metastatic disease,^6^ which reflects the difficulty in diagnosing this entity.

The only curative treatment available is surgery. There are no known effective systemic therapies; the results of chemotherapy and radiotherapy have been disappointing so far.^22^

The recommended surgical approach in these cases is *en bloc* resection,^4^, ^6^ which includes the removal of the tumor as well as the ipsilateral thyroid lobe, since this proved to be the strategy with the lowest recurrence and mortality rates.^5^, ^9^ In spite of these recommendations, local resection of parathyroid gland is still the most commonly performed procedure,^5^ probably because malignancy is not suspected before surgery in most cases.

The diagnosis of parathyroid carcinoma is generally difficult because clear diagnostic markers are still lacking. The presentation is highly variable, sometimes overlapping with the one of parathyroid adenomas.^23^ However, there are several clinical, analytical, and imaging features suggesting malignancy, and these should always be considered before surgery. It is essential to identify the patients at greater risk among those with primary hyperparathyroidism, in order to provide them the most suitable surgical treatment, and not compromise their prognosis.^6^

Parathyroid carcinomas present in most cases with more severe clinical manifestations than adenomas, including concomitant kidney and bone disease,^7^, ^21^, ^24^ as in this case report. This kind of presentation should always raise the suspicion of malignancy.^4^, ^18^, ^23^ Parathyroid carcinomas are usually bigger and more metabolically active than adenomas, in a way that most cases are associated with extremely elevated serum calcium and PTH levels (total calcium levels higher than 3.0‐3.5 mmol/L^5^, ^21^ and PTH levels at least 10 times higher than normal).^21^, ^25^ Nevertheless, cutoff values for diagnosing malignancy are not established and significant overlap exists between the two entities.^21^ Also, most lesions are larger than 3 cm. The detection of a palpable neck mass is highly suggestive of carcinoma, but is neither sensitive nor specific for carcinoma.^4^, ^20^

This diagnostic uncertainty was recently approached in a Consensus Statement from the first European Society of Endocrinology Workshop (PARAT). However, they have considered that patients who present with a palpable mass, a serum total calcium level higher than 3.5 mmol/L, markedly elevated PTH levels, severe renal and/or bone disease and/or laryngeal nerve palsy should be suspected of harboring a parathyroid carcinoma.^26^

Nonfunctioning parathyroid carcinomas with normal serum calcium are very rare. In these cases, diagnosis is often made at more advanced stages, due to mass effect symptoms or local invasion.^12^, ^18^ The finding of dysphonia, due to laryngeal recurrent nerve invasion and palsy, strongly suggests malignancy, mainly if there is no past history of neck surgery.^6^, ^23^

Personal or familiar history of hyperparathyroidism or genetic syndromes associated with parathyroid carcinoma, like HPT‐JT and MEN1, should also raise clinical suspicion.^6^ Although the association of parathyroid and thyroid disorders is quite common in the context of MEN1 or in nonsyndromic cases, coexistence of parathyroid carcinoma and thyroid carcinoma is rare and no relationship has been found between these two entities.^27^ It has been proposed an association between parathyroid adenomas and toxic thyroid nodules, but this association was never established for parathyroid carcinomas.^27^

Imaging tests also do not allow to make a definite diagnosis of malignancy, except in cases of local invasion or metastatic disease, but they can be highly informative.^6^ The most commonly performed examinations are neck ultrasound and ^99m^Tc‐sestamibi scintigraphy. Neck ultrasound is the most frequently used technique to locate the tumor in cases of primary hyperparathyroidism.^23^ Some morphological features, besides lesion size, can suggest malignancy (Table 2). Recently, Liu et al^28^ proposed the ratio between the lesion’s maximum diameter and minimum diameter <1.86 as another important predictor of parathyroid carcinoma. ^99m^Tc‐sestamibi scintigraphy is not useful in the characterization of the lesion, unless distant metastases are present.^6^

**Table 2:** Features of neck ultrasound suggestive of parathyroid malignancy^26^, ^43^, ^44^

Irregular margins
Lesions not deformable by compression with the ultrasound probe
Calcifications
Cysts^a^
Presence of radial intralesional blood vessels
Heterogeneous echogenicity
Infiltration
Ratio between the lesion's maximum diameter and minimum diameter < 1.86

^a^ Cysts can also be present in adenomas.

The third‐/second‐generation PTH ratio is a new tool trying to distinguish carcinoma from adenoma.^6^ Amino‐PTH is a type of PTH recently described that reacts with the third generation of PTH kits but not with those of second generation. This type of PTH represents less than 10% of circulating PTH in healthy individuals; therefore, the normal third‐/second‐generation PTH ratio is less than 1. On the other hand, in the case of parathyroid carcinoma, amino‐PTH is hypersecreted and the ratio inverts to greater than 1.^29^ This can be used as a tumor marker of parathyroid carcinoma, with a sensitivity of 78.5% and a specificity of 98.9% among patients with primary hyperparathyroidism.^29^, ^30^, ^31^

During surgery, documentation of invasion of contiguous structures is highly suggestive of malignancy.^32^ However, in most cases it is not possible to establish the diagnosis of malignancy intraoperatively.^1^ Some findings may be useful though; besides tumor size,^1^ tumor characteristics are also important, as carcinomas are usually white or grayish due to a dense and thick fibrous capsule and have a petrous consistency.^9^, ^19^, ^32^ Conversely, typical adenomas are soft, round, or oval‐shaped and have reddish or brownish coloration.^9^, ^33^ In the case described, the lesion observed during surgery did not have the macroscopic features of parathyroid carcinoma so the surgical team decided to perform a conservative parathyroid resection.

Parathyroid carcinoma can present with a wide range of clinical pictures, even as normocalcemic hyperparathyroidism.^34^ Thus, the possibility of parathyroid carcinoma must be accounted even in the absence of the typical signs, if intraoperative findings are suggestive of carcinoma. In this case, an *en bloc* resection should also be performed.^34^

The diagnosis of parathyroid carcinoma can only be confirmed after histopathological examination. However, as in other endocrine neoplasias, the histological criteria of malignancy are often difficult to define and identify.^18^, ^23^ Therefore, it is critical that the histopathological analysis be undertaken by an experienced pathology team. In some cases, the diagnosis is clear, especially if there is invasion of adjacent tissues, diffuse cellular atypia, and readily identifiable mitotic figures, but in most cases, the diagnosis is only possible when some typical features of malignancy congregate.^10^ In 1973, Schantz and Castleman defined a combination of criteria highly suggestive of malignancy, including the presence of thick fibrous bands with trabecular architecture, the evidence of mitotic activity of tumoral cells, and the presence of vascular and capsular invasion.^19^ However, none of these features is sufficiently sensitive or specific to exclude or confirm the diagnosis, as they can also be present in adenomas.^6^, ^35^, ^36^

The histological criteria used at our hospital at the time are described in Table 1 (adapted from ^10^). This classification includes a category of intermedium risk (atypical adenomas) that includes cases with some but inconclusive features of malignancy.^10^ In the case described, the parathyroid lesion had a trabecular pattern, but revealed low mitotic index, no necrosis, and a thin capsule surrounding the lesion on the periphery; therefore, it was considered to be a parathyroid neoplasm of uncertain malignant potential or an atypical adenoma. Close surveillance is needed, as some of these cases develop recurrence or metastasis.^10^

Other techniques have been studied in an attempt to improve diagnostic accuracy, such as immunohistochemistry.^6^, ^23^ The marker of proliferation Ki67 may be useful; however, because there can be overlap, it cannot be used as the sole criteria for the differential diagnosis between parathyroid carcinoma and adenoma.^37^ Parathyroid carcinomas can also hyperexpress galanin‐3 and lose immunoreactivity to parafibromin and RB‐1. These markers, especially if combined, can be useful to distinguish between benign or malignant disease.^38^

Bisphosphonates are first‐line therapy to treat hypercalcemia related to parathyroid carcinoma; in case of hypercalcemia refractory to bisphosphonates, the addition of a calcimimetic drug (eg, cinacalcet) may be beneficial. Calcimimetic drugs reduce PTH secretion by increasing the sensitivity of the calcium‐sensing receptor.^39^ For patients with parathyroid carcinoma who have hypercalcemia refractory to bisphosphonates and cinacalcet, denosumab is an option. Denosumab is a monoclonal antibody that binds to the receptor activator of nuclear factor‐kappa B ligand, inhibiting bone resorption.^40^ In some case reports, it effectively controlled hypercalcemia, in higher doses than those used for osteoporosis.^40^, ^41^, ^42^

Recently, it has been raised the possibility that treatment with drugs used in other tumors, directed to mutations of PIK3CA or MTOR or amplification of CCND1, can also be used in metastasized parathyroid carcinoma, as these mutations are also frequent in this neoplasia. Nevertheless, clinical studies are necessary to validate this option.^15^

In the case report previously described, despite some clinical and analytical features pointing to carcinoma (male gender, age, very high serum calcium and PTH levels and concomitant bone and kidney disease), the surgical team decided to perform an excision of the affected parathyroid gland and not an *en bloc* resection, given the intraoperatively macroscopic aspect of parathyroid. However, given the high preoperative suspicion of carcinoma, an *en bloc* resection should have been considered. The histopathological examination showed no clear diagnostic criteria of malignancy, though some suspicious features were present. Taking into account that no clear pathological features of malignancy were present and pondering the benefits and risks inherent to a new surgery, it was decided not to perform another surgical intervention and keep long‐term close surveillance at the Endocrinology Outpatient Clinic.

## 4 CONCLUSION

Parathyroid carcinoma is a rare entity, difficult to distinguish from adenoma, which is the main cause of primary hyperparathyroidism. Clinical suspicion prior to surgery is critical as more aggressive surgical resection leads to an improved chance of cure of these patients. The most suspicious features of parathyroid carcinoma are PTH at least 10 times higher than normal, serum calcium higher than 3.5 mmol/L, parathyroid lesion measuring more than 3 cm in its highest dimension, and concomitant severe kidney and bone diseases.

With this case report, we aim to emphasize the challenges in the diagnosis of parathyroid carcinomas, as well as the need for regular and long‐term surveillance of patients with primary hyperparathyroidism and some suspicious features due to the risk of local or metastatic relapse, even if a definite diagnosis of malignancy is not made.

## CONFLICT OF INTEREST

The authors have no conflicts of interest to declare.

## AUTHOR CONTRIBUTIONS

Catarina A. Pereira and Susana Garrido: collected data, made the bibliographic review, and wrote the manuscript. Cláudia Amaral, Olinda Lima, and Helena Cardoso: contributed to the discussion and reviewed the manuscript.
